# Mental Health Status of University Students and Working Professionals during the Early Stage of COVID-19 in Bangladesh

**DOI:** 10.3390/ijerph19116834

**Published:** 2022-06-02

**Authors:** Muhammad Mainuddin Patwary, Mondira Bardhan, Asma Safia Disha, Md Pervez Kabir, Md. Riad Hossain, Md Ashraful Alam, Md. Zahidul Haque, Sharif Mutasim Billah, Matthew H. E. M. Browning, Russell Kabir, Sarya Swed, Sheikh Shoib

**Affiliations:** 1 Environment and Sustainability Research Initiative, Khulna 9208, Bangladesh; mondirabardhan.22@gmail.com (M.B.); asmasafia.disha@ugent.be (A.S.D.); mkabi086@uottawa.ca (M.P.K.); rtr.zahid@gmail.com (M.Z.H.); shakib8376@gmail.com (S.M.B.); 2 Environmental Science Discipline, Life Science School, Khulna University, Khulna 9208, Bangladesh; 3 Institute of Disaster Management, Khulna University of Engineering & Technology, Khulna 9203, Bangladesh; riad.hossain@idm.kuet.ac.bd; 4 Department of Global Health Policy, Graduate School of Medicine, The University of Tokyo, Tokyo 113-0033, Japan; aalam@m.u-tokyo.ac.jp; 5 Tokyo Foundation for Policy Research, Tokyo 106-6234, Japan; 6 Department of Parks, Recreation and Tourism Management, Clemson University, Clemson, SC 29634, USA; mhb2@clemson.edu; 7 School of Allied Health, Faculty of Health, Education, Medicine and Social Care, Anglia Ruskin University, Chelmsford, Essex CM1 1SQ, UK; russell.kabir@aru.ac.uk; 8 Faculty of Medicine, Aleppo University, Aleppo 6458+JPC, Syria; saryaswed1@gmail.com; 9 Department of Psychiatry, Jawahar Lal Nehru Memorial Hospital (JLNMH), Srinagar 190003, India; sheikhshoib22@gmail.com

**Keywords:** COVID-19, mental health, university students, working professionals, Bangladesh

## Abstract

A novel coronavirus disease known as COVID-19 has spread globally and brought a public health emergency to all nations. To respond to the pandemic, the Bangladesh Government imposed a nationwide lockdown that may have degraded mental health among residents, in particular, university students and working professionals. We examined clinically significant anxiety levels with the Generalized Anxiety Disorder (GAD-7) scale and perceived stress levels with the Perceived Stress Scale (PSS-4) in an online cross-sectional study with 744 adults. Approximately 70% of respondents were afflicted with clinically significant anxiety levels, and more than 43.82% were afflicted with moderate or high perceived stress levels. Multivariate logistic regression models showed that postgraduates (OR = 2.78, 95% confidence interval [CI] = 1.03–8.75, *p* < 0.05) were more likely to experience anxiety than their student counterparts. No such differences emerged for working professionals, however. Living with family members compared to living alone was a risk factor for perceived stress among working professionals (OR = 4.05, 95% CI = 1.45–11.32, *p* < 0.05). COVID-19 stressors such as financial hardship (OR = 1.84, 95% CI = 1.11–3.05, *p* < 0.05) and worries of family members’ health (OR = 1.84, 95% CI = 1.12–2.99) were risk factors for anxiety among students. Questionable social media news exposure (OR = 2.99, 95% CI = 1.13–7.92, *p* < 0.05) contributed to the development of mental stress among working professionals. These findings confirm that effective initiatives and proactive efforts from concerned authorities are necessary to cope with the mental health correlates of the COVID-19 pandemic, including in developing contexts such as Bangladesh.

## 1. Introduction

A novel coronavirus disease with flu-like symptoms was recognized in Wuhan, China, in December 2019 [1]. Therefore, in the beginning, Wuhan was the epicenter of the disease [2]. However, the disease spread quickly throughout China and many other countries [3]. Subsequently, on 11 March 2020, the World Health Organization (WHO) announced that the novel coronavirus outbreak was a pandemic and global public health emergency that caused an illness known as COVID-19 [4]. As of 25 July 2021, more than 190 million diagnosed cases with 4.15 million deaths had been reported [5]. To prevent further transmission, a large number of people in affected countries maintained quarantine in their homes to abide by nationwide policies [6]. This prolonged confinement brought widespread concern for the psychological challenges of the pandemic [7]. Earlier studies have shown that lockdown has substantially impacted people’s psychological well-being [8,9].

On 8 March 2020, Bangladesh reported its first confirmed case of COVID-19. The country confirmed its first death from COVID-19 on 18th March [10]. To prevent community transmission, the government declared a nationwide lockdown with the strict prohibition of inter-district movement, economic suspension except for essential services, and cessation of all social gatherings starting on 26 March 2020 [11]. However, many residents did not maintain a proper social distance, which resulted in an increasing rate of infection. Consequently, Bangladesh reported more than 1.19 million infected cases, with 19,779 deaths, as of 27 July 2021 [12]. 

Broadly speaking, the COVID-19 pandemic has caused massive changes in the daily lives of people. The rapid rise of daily cases, false news in the media, shortage of medical equipment, and uncertainty has resulted in fear, anxiety, mental disorders, and even suicidality among the general public, especially younger generations [13,14,15,16]. Notably, Mamun & Griffiths (2020) found that suicidal cases of Bangladeshi residents increased due to fear of COVID-19 [17]. Similar occurrences have been reported in India and Iran [14,18].

Like other countries, Bangladesh implemented a closure of educational institutions to slow the spread of cases [11]. This long-term closing of educational institutions interrupted academic routines, caused financial losses among families, decreased personal space in residences, increased the risk of infection among family members, and decreased security of employment; collectively, these impacts led to a wide range of psychological consequences among university students [1,19]. In addition, universities introduced online or virtual learning programs and assessments to continue higher education. However, rural students had trouble with technological adoptions due to insufficient internet connectivity and access to computers, which further contributed to their emotional burden [20].

Another vulnerable group during COVID-19 was working professionals, and in particular, frontline workers. Doctors, nurses, other medical staff, pharmacists, bankers, law enforcement officers (e.g., police), and some government employees were in close contact with COVID-19-infected patients. As a consequence, these populations were more likely to be exposed to infections that induced fear, worry, and stress [21,22,23]. Simultaneously, uncertainty around employment and financial hardship contributed to the upheaval of mental health [24].

Given these situations, it is important to analyze university students and working professionals’ mental health during the COVID-19 pandemic to inform psychological interventions. Numerous studies have documented anxiety disorders among general populations in China [4], Iran [25], and Bangladesh [26]; psychological disorders in frontline healthcare professionals in China [27], Bangladesh [21,28,29], and medical students in China [30]; psychological impacts on the elderly and university students in China [19,31]; and mental health and well-being concerns among university students in Bangladesh [32,33,34,35]. These studies have also documented potential risk factors for poor mental health, including gender, age, socioeconomic status, occupation, residency, and COVID-19 symptoms [1,36]. More specifically, Islam et al. (2020) found that sociodemographic conditions, including gender, age, educational level, marital status, and employment status, were risk factors for panic and anxiety among people during COVID-19 [35]. Similarly, a psychological study among non-infected populations during the SARS outbreak found that younger ages and people feeling more self-blame showed increased psychiatric morbidities [37]. A study in Poland found that students with higher levels of depression during COVID-19 were more likely to experience temporomandibular joint disorders (TMDs), headaches, and shoulder girdle pain [38]. Meanwhile, working professionals were required to offer emergency services throughout the lockdown time, putting them at risk of virus infection. The situation remains particularly concerning for healthcare workers who have had direct contact with individuals infected with COVID-19. As a result, hospitals were forced to put their staff in quarantine or isolation, which affected the mental health of healthcare professionals [39]. Such factors as quarantine status may have had some influence on the mental health of working professionals.

Psychiatric problems often manifest in early adulthood, making young adults particularly susceptible to these disorders. Previous studies document higher rates of psychiatric symptoms (e.g., depressive symptoms, anxiety symptoms, and suicide risk) among college students than other populations [40,41] due to stress from schoolwork, uncertainties about future employment, and other causes [42,43]. Furthermore, this group might be increasingly prone to psychological distress during the pandemic. Essential working professionals (e.g., healthcare workers) who had to travel to work and experience the risk of infection, in addition to having to treat COVID-19 patients with insufficient information, have also been placed under psychological distress during the pandemic [39]. Direct comparison between students and at-risk working professionals is limited yet would provide insights into the relative impacts of the pandemic on these two vulnerable populations.

To summarize, people in Bangladesh have already suffered from a mental health crisis. Little research has been conducted on university students and working professionals in Bangladesh to evaluate mental health during the government-imposed lockdown period. A detailed study on the psychological status of these two groups and associated risk factors of poor mental health is a prerequisite to mitigate future negative psychological outcomes (e.g., major depressive disorder, post-traumatic stress disorder). Therefore, this study aimed to estimate the prevalence and associated risk factors for anxiety and perceived stress during the COVID-19 lockdown among university students and working professionals in Bangladesh using the Generalized Anxiety Disorder (GAD-7) and Perceived Stress Scale (PSS-4) screening tools.

## 2. Materials and Methods

### 2.1. Study Design and Participants

An online survey was carried out in the starting phase of lockdown from 17 April 2020 to 1 May 2020. Target participants were students and working professionals of Bangladesh aged over 18. Students enrolled in any university in Bangladesh and working professionals across a wide range of professions (i.e., government, non-government, healthcare, banking, and business) were eligible to participate. The questionnaire was developed in English and translated into the local language (Bangla). To collect the survey data, a snowball sampling method was used. We sent out a structured online questionnaire through social networks available to the research team (e.g., Facebook, WhatsApp, and Instagram). We requested people complete the questionnaire and share it with their own online social networks. Complete data from 544 students and 200 working professionals (n = 744) were retrieved and included in the final analysis. The research followed the principles enunciated by the Declaration of Helsinki of 1964 and its subsequent amendments [44]. The ethics of the study was also approved by the Institute of Disaster Management of Khulna University of Engineering & Technology, Khulna, Bangladesh as exempt. Electronic informed consent was obtained from all the respondents prior to their participation.

### 2.2. Measures

#### 2.2.1. Mental Health 

The Generalized Anxiety Disorder (GAD-7) [45] assessed the anxiety levels of respondents. This is a well-approved screening tool with excellent validity and reliability in our sample (Cronbach’s α = 0.911) and previous research [4,19]. The GAD-7 includes seven items that measure the frequency of participants’ symptoms of suffering over the past two weeks. Participants self-report their symptom severity on a 4-point Likert-type scale from 0 (not at all) to 3 (almost every day). By summing all items, a summary score is generated. The overall score varies from 0 to 21, with higher values indicating more severe symptomology. Respondents are classified as having little or no anxiety (summary scores of 0–4), mild (5–9), moderate (10–14), or severe anxiety (15–21). A cutoff score of 10 or higher is regarded as a clinically significant degree of anxiety [45].

The Perceived Stress Scale-4 (PSS-4) measured the perceived stress of respondents. This scale is a valid and reliable measure of participants’ feelings of control and confidence in their ability to handle difficult situations over the preceding month [46]. The PSS-4 is a shortened version of the PSS-10 and consists of four items regarding respondents’ ability to exert influence over significant events, confidence in dealing with personal issues, sense of things going their way, and sense of being overwhelmed by mounting challenges [47]. Participants rate their responses on a 4-point Likert-type scale from 0 (never) to 4 (very often) [46]. A summary score is calculated by combining the individual scores. The resulting summary score ranges from 0 to 16. There is no set cut-off point for detecting signs of excessive stress; rather, patients’ scores were compared to a normative value with higher scores indicating that a patient’s capacity to deal with stress was exceeded [48]. In the present study, the PSS-4 internal consistency was acceptable (Cronbach’s alpha = 0.81).

In alignment with prior work, we divided the PSS-4 summary scores by tertile [49]. A score from 9–16 was defined as the highest tertile and indicated high levels of stress. Scores from 6–8 demarcated the second tertile and represented a moderate level of stress. Scores between 0–5 represented the third tertile and a low level of stress. These ranges approximate earlier research that identified PSS-4 scores of ≥6 indicated moderate to high levels of stress and worse patient outcomes [48]. To conform with previous research, we used a threshold of ≥6 to describe patients with greater stress levels than the normative data.

#### 2.2.2. Risk Factors

Based on past research, we analyzed sociodemographics, urbanicity, living status, quarantine status, and frontline service status as potential risk factors for poor mental health. Sociodemographic characteristics included age, gender, and level of education. The level of education was divided into four groups: (1) less than college level and currently enrolled or passed school and higher secondary school level, (2) current undergraduate student, (3) current graduate student, and (4) completed a graduate degree. Urbanicity was self-reported as urban or rural. Living status included three groups: (1) living with family members, (2) living with others who are not family members, and (3) living alone. Quarantine status was determined by asking respondents if they were quarantined or in social isolation. Frontline service status was judged by respondents as active involvement in providing emergency assistance during the COVID-19 lockdown. Many of the working professionals who reported careers in healthcare, pharmacies, bankers, police officers, and government administrative personnel were classified as frontline service providers.

For the current study, five questions related to COVID-19 stress were asked as additional potential risk factors: (1) Are you worried about the financial condition of your family during COVID-19? (2) Are you concerned about your academic delay due to lockdown? (3) Are you worried about being infected with COVID-19 and transmitting it to your family members? (4) Are you weary from spreading false and bad news about COVID-19 on social media? (5) Do you think your career is uncertain due to COVID-19? Each response was recorded as a binary outcome (1 = yes, 0 = no).

### 2.3. Analysis

Sample characteristics were reported with as percentages (%) for categorical data and mean (SD) for continuous data. Chi-square and Kruskal–Wallis tests were used to compare sample characteristics of students and working professionals. To determine risk factors for poor mental health, bivariate correlations and multivariable (adjusted) logistic regression models were conducted with binary outcome variables. These binary variables used standard cutoff values for anxiety (≥10) and perceived stress (≥6) (see 2.2.1. Mental Health Outcomes. Only statistically significant predictors in the correlation analyses were used in the multivariable models. A two-tailed test with a significance level of *p* < 0.05 was judged statistically significant. R software and SPSS statistical software (V 26) were used for all analyses.

## 3. Results

### 3.1. Sample Characteristics

The descriptive statistics of respondents are presented in Table 1. Most were male (58%), 30 years old or less (93%), urban dwellers (87%), and living with family members (76%). Two-thirds of student respondents were undergraduates. Approximately one-third of working professionals had graduate (34%) and postgraduate (35%) degrees. Compared to working professionals, students were more likely to be ≤30 years old (100% vs. 79%) and living with family members (79% vs. 68%). Working professionals were more likely to live in urban areas than students (92% vs. 85%). Nearly one-third (32%, n = 175) of students and working professionals (32% and 28%, respectively) were quarantined during the lockdown period while 88% of working professionals were frontline service providers. The vast majority (81%) reported that they faced financial difficulties during lockdowns. Approximately three-quarters (73%) of students expected academic delays due lockdowns. The vast majority (78%) reported that they were worried about their family members being affected by COVID-19. A substantial portion of respondents reported that questionable social media news exposure (68%) and career uncertainty (72%) were COVID-19 stressors for them.

### 3.2. Mental Health Levels during the COVID-19 Lockdown

Perceived stress and anxiety levels are reported in Table 2. More than two-thirds of respondents showed higher than average levels of perceived stress (70%). Nearly half of respondents reported clinically significant levels of anxiety (44%). Perceived stress and anxiety levels were similar between students and working professionals, with some exceptions. A larger share of working professionals than students reported high perceived stress levels (*p* < 0.05). In contrast, a larger share of students than working professionals reported severe anxiety levels (*p* < 0.05).

### 3.3. Risk Factors for Poor Mental Health during the COVID-19 Lockdown

Table S1 presents the results of bivariate correlations between potential risk factors, anxiety, and perceived stress. Undergraduate students were more likely to show clinically significant levels of anxiety than others (*p* < 0.05). Occupation was significantly associated with both anxiety (*p* < 0.01) and perceived stress (*p* < 0.05). Living with family members was correlated with higher than average perceived stress levels (*p* < 0.05). COVID-19 stressors such as financial hardship, family members’ health, and exposure to questionable social media news exposure were risk factors for anxiety (*p* < 0.01). However, academic delay was negatively associated with anxiety (*p* < 0.01). No COVID-19 stressors predicted perceived stress levels in the entire sample (*p* > 0.05).

Multivariate models run with only the significant risk factors identified in the bivariate correlation analyses are presented in Table 3. Educational level and COVID-19 stressors, including financial hardship, academic delay, and family members’ health, remained risk factors for clinically significant anxiety levels, whereas only living status and questionable social media news exposure remained risk factors for higher-than-average perceived stress levels. Regarding educational status, postgraduate students were nearly three times more likely to show clinically significant anxiety levels than college students (OR = 2.78, 95% CI = 1.03–8.75, *p* < 0.05). No differences in anxiety levels for education status among working professionals were observed. Working professionals were four more likely to show higher than average perceived stress levels if they lived with family members than if they lived alone (OR = 4.05, 95% CI = 1.45–11.32, *p* < 0.05). Students facing financial hardship during the lockdown period were nearly twice as likely to show higher anxiety levels than students who did not face such circumstances (OR = 1.84, 95% CI = 1.11–3.05, *p* < 0.05). In contrast, academic delays were negatively associated with anxiety levels among students (OR = 0.53, 95% CI = 0.35–0.79, *p* < 0.05). Further, students who were worried about their family members being affected by COVID-19 were nearly twice as likely to show clinically significant anxiety levels than those who did not worry (OR = 1.84, 95% CI = 1.12–2.99). Working professionals worried about exposed to questionable social media news were three times more likely to show higher than average perceived stress levels than other working professionals (OR = 2.99, 95% CI = 1.13–7.92, *p* < 0.05). No differences in anxiety among students in regard to questionable social media news exposure were observed.

## 4. Discussion

### 4.1. Summary of the Main Findings

Since December 2019, COVID-19 has spread to many countries, bringing a global public health crisis. The pandemic has caused widespread concerns related to fear, anxiety, and mental distress [50]. As a developing country, Bangladesh faces many challenges associated with the stress and anxiety related to the first wave of the COVID-19 pandemic. The Bangladesh Government has taken measures to prevent further transmission including the closure of educational institutes, country-wide and local lockdowns, regulations of working hours, public safety measures through thermal scanners, and accelerating vaccination development and rollout [11,51,52,53]. However, long periods of lockdown are expected to degrade mental health, particularly among populations most affected by these governmental measures such as students and working professionals [50]. Many studies have investigated the impacts of public health crises on stress, anxiety, and mental illness (i.e., [4,54]). To the best of our knowledge, this is the first study in Bangladesh that compares the mental health status of these university students and working professionals.

We found that more than three in four students and working professionals experienced clinically significant anxiety levels during the early stages of the COVID-19 pandemic in Bangladesh. This finding aligns with several previous studies [1,55]. However, one study by Cao et al., (2020) reported that a much lower proportion (25%) of students showed clinical diagnoses of an anxiety disorder [19]. A possible reason for this difference was that the earlier study used the IES-R scale, which evaluated the psychological effects after an event such as the COVID-19 pandemic [1], whereas we used a generalized anxiety measure not tied to an event. We also found that high ratios of students and working professionals reported moderate to high levels of perceived stress. These findings align with research in Paraguay that showed 78% of residents with similarly high stress levels during the pandemic [56].

The potential cause of anxiety and stress among university students could be related to academic disruptions [57], extra burdens due to virtual classes [58], and uncertainty about their future careers [59]. Further, the rapid rise in the number of daily suspected cases, lack of emergency medical supplies, sensational news, media speculation, and home confinement could have exacerbated students’ psychological disturbances [60,61,62]. Keeping physical distance from others and abstaining from in-person communication with friends for a long period are likely to trigger anxiety and depressive disorders [63]. Such increases in anxiety and depressive symptoms could then trigger perceived stress in people [64]. Furthermore, the lack of adequate exposure to sunlight due to prolonged quarantine could result in decreased levels of serotonin in the body, which could have contributed to emotional disorders [65]. Some studies have found that students with less quarantine time reported more positive psychological behavior [7,66,67]. Beyond the common reasons for increased stress and anxiety, working individuals (government and non-government employees and healthcare workers) encountered several extra burdens related to job insecurity, economic concern, and working from home. The unprecedented financial ramifications as well as the fear of losing employment could have resulted in poor mental health [24]. A recent survey found that three-fourths of respondents reported new mental health problems while working from home, and among them, more than half faced two or more new psychological disturbances during the pandemic [68].

Our study found that educational status may be a risk factor for students developing anxiety during the pandemic. Students at the postgraduate level, in particular thesis-term students, were more likely to develop a clinically significant anxiety levels than others, which has also been reported in earlier research [69]. This finding could be attributed to the interruption of final research and thesis projects resulting in graduation delays that, in turn, had adverse impacts on competitiveness for potential job markets [70]. Additionally, as another measure for preventing the spread of COVID-19, the Government of Bangladesh shut down all the educational institutions as of 18 March 2020, which pushed universities to postpone classes or pursue online education. These changes undoubtedly disrupted academic routines [20] and led to poor mental health among students [57].

Living with family members was another risk factor for developing mental stress among working professionals. Participants who were with their family members during lockdown might have been more worried about family members getting infected by the virus. It is likely that persons who lived alone were better accustomed to isolation, but those who lived with family members (e.g., father, mother, spouse, children, etc.) may have felt more stress and anxiety as a result of the extended contact inside the home during confinement [71]. Further, many working professionals had to return to their workplaces during the pandemic, which may have increased mental stress as a result of their overwhelming concern that their families might get infected. For example, healthcare workers who directly dealt with COVID-19 patients had to go to their workplaces, and were more susceptible to exposure [72]. Therefore, the virus may have been transmitted to family members and contributed to mental stress among the healthcare workers. Some scholars have authenticated this statement by showing positive correlations between perceived risk and stress [73]. Moreover, worrying about getting infected or transmitting the virus to family members could have made workers even more stressed. During the early phases of COVID-19, research found that participants whose family members worked in healthcare were 44% more likely to develop mental illness [74].

Our findings show that COVID-19 stressors, including financial hardship and worry about family members’ health, were positively associated with students’ anxiety levels. Similar findings were reported by Cao et al. (2020) [19]. The loss of family income has an influential role in anxiety among students during the pandemic [75]. A previous study also demonstrated that younger people tend to have heavier financial burdens during crisis periods, leading to elevated anxiety symptoms [76]. The Government of Bangladesh announced a nationwide shutdown on 26 March 2020 that suspended all economic activity, resulting in a lack of income for many households [11]. Because of this, students may have felt anxious about paying their tuition fees. Moreover, many students operated part-time jobs or private tuition while at the campus for financial independence. However, under the lockdown, the inability to pay for tuition entails a loss of regular income and joblessness. This prolonged financial hardship would cause students to worry about the pending fee for their room, aggravating the financial crisis and mental illness [77]. A study suggests that prolonged joblessness is a risk factor for developing mental disorders among young adults [78]. Additionally, younger individuals could be asymptomatic carriers, as reported by Pan et al. (2020); this left students anxious about high risks of infection for older persons in their families [79]. Students living with older family members during the restriction period were likely more prone to psychological suffering for this reason. Another study reported that students who were living with their family members were nearly twice as likely to develop anxiety [35].

Unexpectedly, our findings found a negative association between academic delays and anxiety symptoms among students. This might be because students believed that the lockdown time would not be prolonged further, and they would continue their normal classes. Several universities quickly decided to continue classes in an online format, which could have contributed to reducing session-jam and bringing some relief to students [80].

Finally, working professionals exposed to questionable social media news were more likely to develop high anxiety levels. Because of the lockdown, people appeared to have a long-time connection to internet content and social networking, which was likely to increase mental stress because social media covers sensational news and people share negative and false news through social media [33,81]. A Taiwanese study reported that around 80% of people spent more time on the internet to gain information about the pandemic during lockdown [82]. A study in Bangladesh reported that participants with 4 hours of exposure to social media were 52% more likely to develop anxiety than those with less than two hours of exposure [83].

### 4.2. Strengths and Limitations

This study had several strengths. First, the study examined a nationwide sample of university students as well as working professionals, and data were collected through online platforms to maintain WHO-recommended social distances. Second, the study used a globally validated tool for assessing mental health. Last, the study selected a snowball sampling method over random sampling for data collection in a limited resource setting during the COVID-19 pandemic.

However, this study also had limitations. First, the study did not consider other factors such as marital status, family income, and pre-existing illness that could have impacted mental health. Second, the study involved self-reported data from an online survey that could have been witness to response or self-selection bias. Third, the sample size may not have been representative to the entire country or other settings because only respondents with internet access could participate. Fourth, the data were collected from 17 April 2020 to 1 May 2020, which did not encompass the entire early stages of the COVID-19 pandemic. Last, we could not confidently draw causal relationships between variables from the cross-sectional dataset. Future research should focus on longitudinal studies for making inferences about the psychological effects of the COVID-19 pandemic on students and working professionals.

## 5. Conclusions

This study highlighted the negative psychological effects of the COVID-19 pandemic on university students and working professionals in Bangladesh. A substantial portion of participants reported moderate to high levels of anxiety and perceived stress. Postgraduate level students were at higher risk of developing clinically significant anxiety levels. Respondents living with family members were more likely to suffer from mental stress. COVID-19 stressors such as financial hardship, worries about family members’ health, and worries about questionable social media news were risk factors for poor mental health.

Such high levels of mental distress are alarming and should be addressed vigorously and urgently. Since the duration of the ongoing pandemic remains unknown, mental distress is likely to continue and present immediate and long-lasting concerns. Responsible authorities should impose positive psychological interventions, such as social support, counseling, job security, and easing academic assessment systems. Spending quality time with family members and engaging in physical exercise may be effective mental health measures during this prolonged, global state of emergency.

## Figures and Tables

**Table 1:** Descriptive statistics of respondents (n = 744).

	Sample Characteristics		
Variables	Entire Sample (n= 744)	University Students (n = 544)	Working Professionals (n = 200)
Gender			
Male	432 (58.00)	310 (57.00)	122 (61.00)
Female	312 (42.00)	234 (43.00)	78 (39.00)
Age			
≤30	697 (94.00)	539 (99.00)	158 (79.00)
>30	47 (6.00)	5 (1.00)	42 (21.00)
Education level			
≤College	51 (7.00)		51 (25.00)
Undergraduate	369 (49.00)	362 (67.00)	12 (6.00)
Graduate	221 (30.00)	148 (27.00)	68 (34.00)
Postgraduate	103 (14.00)	34 (6.00)	69 (35.00)
Area of residence			
Urban	645 (87.00)	461 (85.00)	184 (92.00)
Rural	99 (13.00)	83 (15.00)	16 (8.00)
Living status			
With family members	564 (76.00)	429 (79.00)	135 (68.00)
With non-family members	133 (18.00)	89 (16.00)	44 (22.00)
Alone	47 (6.00)	26 (5.00)	21 (10.00)
Quarantine status			
Yes	230 (31.00)	175 (32.00)	55 (28.00)
No	514 (69.00)	369 (68.00)	145 (72.00)
Frontline service provider			
Yes	175 (87.00)	0 (0.00)	175 (87.00)
No	25 (13.00)	0 (0.00)	25 (13.00)
COVID-19 STRESSORS			
Financial hardship			
Yes	602 (81.00)	434 (80.00)	168 (84.00)
No	142 (19.00)	110 (20.00)	32 (16.00)
Academic delay			
Yes	395 (73.00)	395 (73.00)	
No	149 (27.00)	149 (27.00)	
Family members health			
Yes	583 (78.00)	415 (76.00)	166 (84.00)
No	161 (22.00)	129 (24.00)	34 (16.00)
Exposure to questionable social media news			
Yes	505 (68.00)	354 (65.00)	151 (75.00)
No	239 (32.00)	190 (35.00)	49 (25.00)
Career uncertainty			
Yes	538 (72.00)	394 (72.00)	144 (72.00)
No	206 (28.00)	150 (28.00)	56 (28.00)

**Table 2:** Perceived stress and anxiety levels during the COVID-19 lockdown in Bangladesh (n= 744).

	Mental Health Levels			Differences between Students and Professionals	
Variables	Entire Sample	University Students (n= 544)	Working Professionals (n = 200)	χ^2^	*p*-Value
Perceived stress					
Low (0–5)	226 (30.00)	151 (28.00)	75 (38.00)		
Moderate (6–8)	351 (47.00)	270 (50.00)	81 (41.00)		
High (9–16)	167 (23.00)	123 (23.00)	44 (22.00)		
Higher than average (≥6)	518 (70.00)	393 (72.00)	125 (63.00)	4.94	0.026 *
Anxiety					
Normal (0–4)	169 (23.00)	120 (22.00)	49 (24.00)		
Mild (5–9)	249 (33.00)	173 (32.00)	76 (38.00)		
Moderate (10–14)	184 (25.00)	138 (25.00)	46 (23.00)		
Severe (15–21)	142 (19.00)	113 (21.00)	29 (15.00)		
Clinically significant levels (≥10)	326 (44.00)	251 (46.00)	75 (38.00)	4.43	0.035 *

Notes: n (% of sample) reported, * *p* < 0.05.

**Table 3:** Multivariable logistic regression results of associated risk factors of perceived stress and anxiety during COVID-19 lockdown in Bangladesh.

Variables	University Students (n= 544)		Working Professionals (n = 200)	
	Higher than Average Perceived Stress (Model 1)	Clinically Significant Anxiety (Model 2)	Higher than Average Perceived Stress (Model 3)	Clinically Significant Anxiety (Model 4)
Education level				
Postgraduate		2.78 (1.03–8.75) *		1.25 (0.44–3.51)
Graduate		1.88 (0.74–4.79)		0.93 (0.33–2.67)
Undergraduate		1.88 (0.75–4.62)		1.18 (0.36–3.87)
≤College		[Ref.]		[Ref.]
Living status			-	
With family members	1.82 (0.80–4.13)		4.05 (1.45–11.32) *	
With non-family members	2.04 (0.79–5.23)		2.76 (0.88–8.61)	
Alone	[Ref.]		[Ref.]	
Financial hardship				
Yes		**1.84 (1.11–3.05)** *		2.16 (0.76–6.12)
No		[Ref.]		[Ref.]
Academic delay				
Yes		0.53 (0.35–0.79) *		
No		[Ref.]		
Family members health				
Yes		1.84 (1.12–2.99) *		0.41 (0.14–1.19)
No		[Ref.]		[Ref.]
Questionable social media news exposure				
Yes		1.18 (0.78–1.78)		2.99 (1.13–7.92) *
No		[Ref.]		[Ref.]

Notes: Odds ratios (95% confidence intervals) shown; Cutoffs for binary mental health outcomes included ≥6 for perceived stress (PSS-4), and ≥10 for anxiety on the GAD-7; Ref. = reference value; Significant findings (* *p* < 0.05) shown in bold.

## Data Availability

Data generated in this study is available by contacting the first corresponding author, Muhammad Mainuddin Patwary, if reasonably requested.

## Supplementary Materials

The following supporting information can be downloaded at: https://www.mdpi.com/article/10.3390/ijerph19116834/s1, Table S1: Bivariate correlation of risk factors for poor mental health during the COVID-19 lockdown in Bangladesh (n = 744).
