# Supplementary material for: Mental Health Status of University Students and Working Professionals during the Early Stage of COVID-19 in Bangladesh
Source: Int J Environ Res Public Health. 2022 Jun 2;19(11):6834. doi: 10.3390/ijerph19116834 (PMC9180371; doi:10.3390/ijerph19116834)
Supplement: Supplementary file 1 [file ijerph-19-06834-s001.zip › ijerph-1704160-supplementary.pdf]

Table S1. Bivariate correlation of risk factors for poor mental health during the COVID-19 lockdown in Bangladesh (N=744).

	1	2	3	4	5	6	7	8	9	10	11	12	13	14	15
1. Gender	1	-0.01	0.04	-0.15**	-0.04	-0.02	-0.05	0.03	-0.01	0.03	-0.06	-0.07	-0.04	0.03	-0.04
2. Age		1	0.18**	-0.04	0.37**	0.31**	0.01	-0.03	0.06	-0.03	0.04	0.04	0.01	0.00	-0.02
3. Education			1	-0.02	0.32**	0.27**	-0.01	-0.08*	0.02	-0.01	0.04	0.04	0.03	0.16*	0.00
4. Residence				1	-0.09**	-0.08*	0.01	0.01	-0.06	-0.06	-0.04	0.02	-0.01	0.02	0.03
5. Occupation					1	0.91**	0.00	-0.04	0.05	0.03	0.07	-0.04	0.00	.099**	0.07*
6. Living status						1	0.00	-0.01	0.13**	-0.11*	0.02	.076*	-0.01	0.03	0.01*
7. Quarantine							1	-0.02	-0.03	-0.11**	-0.08*	-0.07*	-0.02	0.01	-0.04
8. Frontline service[1]								1	0.07	0.03	0.07	.083*	-0.02	-0.02	0.00
9. Financial hardship									1	-0.11*	0.37**	0.28**	-0.01	0.16**	0.01
10. Academic delay[2]										1	-0.05	-0.03	.11*	-0.14**	0.01
11. Family health											1	.44**	-0.02	0.14**	-0.04
12. Social media exposure												1	-0.04	0.13**	-0.02
13. Career uncertainty													1	0.05	0.07
14. Anxiety[3]														1	-0.02
15. Stress[4]															1

**correlation is significant at 0.01 level (2-tailed). *correlation is significant at 0.05 level (2-tailed). [1] Frontline service: analysis was done only for working professionals; [2]Academic delay: analysis was done only for student; [3]Anxiety was measured using Generalized Anxiety Disorder-7 (GAD-7) scale and cutoff ≥10 considered as anxiety; [4]Perceived stress was measured using Perceived Stress Scale (PSS-4) scale and cutoff ≥6 considered as higher than average stress.
